# Understanding the challenges, unmet needs, and expectations of mucopolysaccharidoses I, II and VI patients and their caregivers in France: a survey study

**DOI:** 10.1186/s13023-022-02593-2

**Published:** 2022-12-23

**Authors:** Nathalie Guffon, Delphine Genevaz, Didier Lacombe, Eliane Le Peillet Feuillet, Pascale Bausson, Esther Noel, François Maillot, Nadia Belmatoug, Roland Jaussaud

**Affiliations:** 1 Reference Center for Inherited Metabolic Disorders of Lyon, (CERLYMM), Hospices Civils de Lyon, 69677 Bron, France; 2 Vaincre Les Maladies Lysosomales, 91300 Massy, France; 3 Medical Genetics Unit, University Hospital of Bordeaux, INSERM U1211, 33076 Bordeaux, France; 4 Sanofi, 94250 Gentilly, France; 5 Study Department, AplusA Company, 92641 Boulogne Billancourt, France; 6 University Hospital of Strasbourg, BP 426, 67100 Strasbourg, France; 7 Department of Internal Medicine, Regional University Hospital of Tours, 37000 Tours, France; 8 Reference Center of Lysosomal Diseases, Beaujon Hospital, 92110 Clichy, France; 9 Department of Internal Medicine and Clinical Immunology, Nancy University Hospital, 54500 Vandoeuvre-Les-Nancy, France

**Keywords:** Caregiver, Disability, Mucopolysaccharidoses, Quality of life, Qualitative survey

## Abstract

**Background:**

Mucopolysaccharidoses (MPS) are a group of inherited lysosomal storage diseases caused by defective enzyme activity involved in the catalysis of glycosaminoglycans. Published data on adult patients with MPS remains scarce. Therefore, the present qualitative survey study was aimed at understanding knowledge of the disease, unmet needs, expectations, care, and overall medical management of adult/adolescent patients with MPS I, II and VI and their caregivers in France.

**Results:**

A total of 25 patients (MPS I, n_p_ = 11; MPS II, n_p_ = 9; MPS VI, n_p_ = 5) were included and about 36 in-depth interviews (caregivers alone, n_c_ = 8; patients-caregiver pair, n_c+p_ = 22; patients alone, n_p_ = 6) were conducted. Except one (aged 17 years), all patients were adults (median age: 29 years [17–50]) and diagnosed at median age of 4 years [0.4–30], with mainly mothers as caregivers (n_c_ = 16/19). Patients were classified into three groups: Group A, Patients not able to answer the survey question because of a severe cognitive impairment (n_p_ = 8); Group B, Patients able to answer the survey question with low or no cognitive impairment and high motor disability (n_p_ = 10); and Group C, Patients able to answer the survey question with low or no cognitive impairment and low motor disability (n_p_ = 7). All groups were assessed for impact of disease on their daily lives based on a scale of 0–10. Caregivers in Group A were found to be most negatively affected by the disease, except for professional activity, which was most significantly impacted in Group B (4.7 vs. 5.4). The use of orthopaedic/medical equipments, was more prevalent in Groups A and B, versus Group C. Pain management was one of the global unmet need expressed by all groups. Group A caregivers expected better support from childcare facilities, disability clinics, and smooth transition from paediatric care to adult medicine. Similarly, Group B caregivers expected better specialised schools, whereas Group C caregivers expected better psychological support and greater flexibility in weekly infusion schedules for their patients.

**Conclusions:**

The survey concluded that more attention must be paid to the psychosocial status of patients and caregivers. The preference for reference centre for follow-up and treatment, hospitalizations and surgeries were evident. The most significant needs expressed by the patients and caregivers include better understanding of the disease, pain management, monitoring of complications, flexibility in enzyme replacement therapy, home infusions especially for attenuated patients, and improved transitional support from paediatric to adult medicine.

**Supplementary Information:**

The online version contains supplementary material available at 10.1186/s13023-022-02593-2.

## Background

Mucopolysaccharidoses (MPS) are a group of rare, inherited, lysosomal storage disorders triggering multisystemic clinical manifestations [1–3]. The defective lysosomal enzyme activity results in the accumulation of glycosaminoglycans (GAGs) such as heparan sulfate (HS), chondroitin sulfate (CS), keratan sulfate (KS), and dermatan sulfate (DS) [1, 2].

Seven types of MPS have been described (MPS I, II, III, IV, VI, VII, and IX) in the literature [1, 2]. A recent retrospective study in the United States (US) reported that MPS I, II, and III had higher incidence (0.26/100,000 live births), as compared to MPS VI (0.04/100,000) [1]. However, there is a paucity of such epidemiological data on MPS in France. In a recent 2021 review paper summarizing epidemiological data of MPS in 33 countries [2], the only study included from France is a 2010 epidemiological study reporting the incidence of only MPS III, which was 0.68/100,000 live births [4].

All MPS types have a clinical spectrum, i.e., neuronopathic and non-neuronopathic. The severe end of the spectrum for MPS I and MPS II with rapidly progressing patients comes under neuronopathic phenotype (involvement of the brain) whereas the milder end of the spectrum includes non-neuronopathic patients for MPS I, II and MPS VI with slowly progressing symptoms. Usually, patients in both MPS I and II accumulate common GAGs (HS and DS), therefore, patients share some of the characteristic phenotypical manifestations, such as short stature due to skeletal disease, inguinal and umbilical hernias, hydrocephalus, hearing loss, coarse facial features, protruded abdomen with hepatosplenomegaly, and varying degree of cognitive dysfunction based on HS/DS ratio [5]. Clinical presentation of MPS VI is similar to MPS I without neurocognitive impairment based on only DS accumulation. The severe phenotype is characterized by more severe dysostosis multiplex with a final mean height of 1.15 m [6]. Earlier, the life expectancy of patients with severe phenotypic MPS was short, due to the unavailability of diagnostic methods, and multi-disciplinary care, and hence, very limited patients survived beyond childhood [7]. However, with improved care and the emergence of new therapies, patients with MPS I, MPS II, and VI have shown a trend towards longer survival over recent decades (1990–2010s), leading to an increase in the population of older MPS patients. Considering that this trend towards longer survival will continue to grow, MPS is expected to eventually become a chronic medical issue in the future [7, 8].

The current treatment options available for MPS, such as haematopoietic stem cell transplantation (HSCT) and/or enzyme replacement therapy (ERT), alleviate symptoms of the disease but do not constitute a definite cure. The ERT improves mobility and pulmonary function, reduces the risk of organ damage, and substantially improves the QoL for most patients with MPS [9–13]. However, recombinant enzyme is inefficient in crossing the blood–brain barrier, hence there are no benefits of ERT in preventing or improving cognitive decline. Also, the effect of ERT on bones, cartilage and cardiac valves is very limited [14]. Consequently, patients with MPS require frequent care and assistance from parents, siblings, and other family members [15]. The physical, emotional, social, and financial impact of MPS not only affects patients but also their families and caregivers providing long-term care [16]. Therefore, it is imperative to understand their life course, unmet needs, challenges in care and develop best practices and follow-up protocols for caregivers and patients struggling with the disease [17].

The most effective approach to explore about the experience and challenges in the daily life of both patients and caregivers living with a rare condition is qualitative survey studies conducted via in-depth interviews [17, 18]. Particularly in rare diseases such as MPS, a qualitative study can allow a comprehensive understanding of the diversity of attitudes, behaviors, and experiences of an individual living with a rare disorder [19, 20].

The burden of MPS in France has not been thoroughly investigated, except in a retrospective observational study, which evaluated the MPS II burden, organization of clinical care, and effects of idursulfase treatment on the disease in 52 patients in France [21]. Therefore, this French national survey study was conducted to understand the overall management and perception of adolescent and adult patients with MPS I (Hurler, Hurler-Scheie, Scheie syndrome), MPS II (Hunter syndrome), and MPS VI (Maroteaux-Lamy syndrome).

This survey study aims to enable adolescent/adult patients and their caregivers to share their disease-related experience and how it impacts their quality of life on a daily basis. Additionally, the survey intends to provide an opportunity to express their unmet needs, expectations, and areas for improvement in coping with the disease. Such in-depth information will hopefully assist healthcare system in improving the quality of care for patients and their caregivers.

## Results

### Patient and caregiver population

A total of 36 in-depth interviews (patient with caregiver pair, n_p+c_ = 22; patient alone, n_p_ = 6; caregivers alone representing patients with severe cognitive impairment form, n_c_ = 8) were conducted for 25 patients (MPS I, n_p_ = 11; MPS II, n_p_ = 9; MPS VI, n_p_ = 5). The demographic details of the patients, as well as the caregivers, are summarized in Table 1 whereas demographic details of each individual patient have been summarized in Additional file 1: Table S1. Except one patient (aged 17 years), all patients were adults with a median age of 29 years [17–50] and diagnosed at median age of 4 years [0.4–30], whereas caregivers were mainly mothers (n_c_ = 16/19), mostly under 60 years of age (n_c_ = 13/19), and having a full-time or part-time professional activity.

**Table 1:** Demographic profile of patients and caregivers

Patient profile	Patients with severe cognitive impairment (n = 8)		Patients with low or no cognitive impairment (n = 17)		
	MPS I (n_p_ = 3)	MPS VI (n_p_ = 5)	MPS I (n_p_ = 8)	MPS II (n_p_ = 4)	MPS VI (n_p_ = 5)
*Gender*					
Men	2	5	2	4	4
Women	1	0	6	0	1
*Age*					
17–20 years	1	2	2	0	1
21–34 years	1	2	2	3	2
29–50 years	1	1	4	1	2
*Education*					
Preschool	2	3	0	0	0
Primary school	1	2	0	0	0
High school	0	0	1	0	0
Vocational certificate	0	0	2	0	2
Higher education	0	0	5	3	3
Disability-friendly class	0	0	0	2	0
Access to specialised classes	2	1	0	0	0
*Current living location*					
Complete dependency on external institutes*	3	4	0	0	0
Complete dependency on parenteral home	0	1	3	2	2
Living alone	0	0	4	1	3
Living in a shared home	0	0	1	1	0
*Age of patient at diagnosis (years)*					
< 1 year	1	0	4	0	0
1–10	2	5	4	9	3
> 10	0	0	3	0	2
Other family members diagnosed with MPS	0	0	1	1	2

Caregiver profile	Patients with severe cognitive impairment (n_c_ = 8)		Patients with low or no cognitive impairment forms (n_c_ = 17)		
*Relationship with patient*					
Father	1		1		
Mother	7		9		
Spouse	0		1		
*Age of the parent interviewed (years)c*					
< 50	2		6		
50–60	4		1		
61–70	0		2		
≥ 71	0		2		
ND	2		0		
*Professional activity*					
Yes (Part time/Full time)	7		5		
No (Retired)	1		4		
ND	0		2		
*Marital status*					
Married	3		6		
Separated/widower	5		3		
ND	0		2		
*Number of children*					
1	3		3		
2	4		6		
3	1		2		

*MPS* Mucopolysaccharidoses, *n*_*c*_ Number of caregivers, *n*_*p*_ Number of patients, *ND* Not disclosed

^*^External institutes refer to medical foster care, sheltered workshops, day care centres, disability clinics, and medical professional institutes

### Knowledge about the disease expressed by patients and caregivers

#### Knowledge gained from personal experiences

**Fig. 1:**
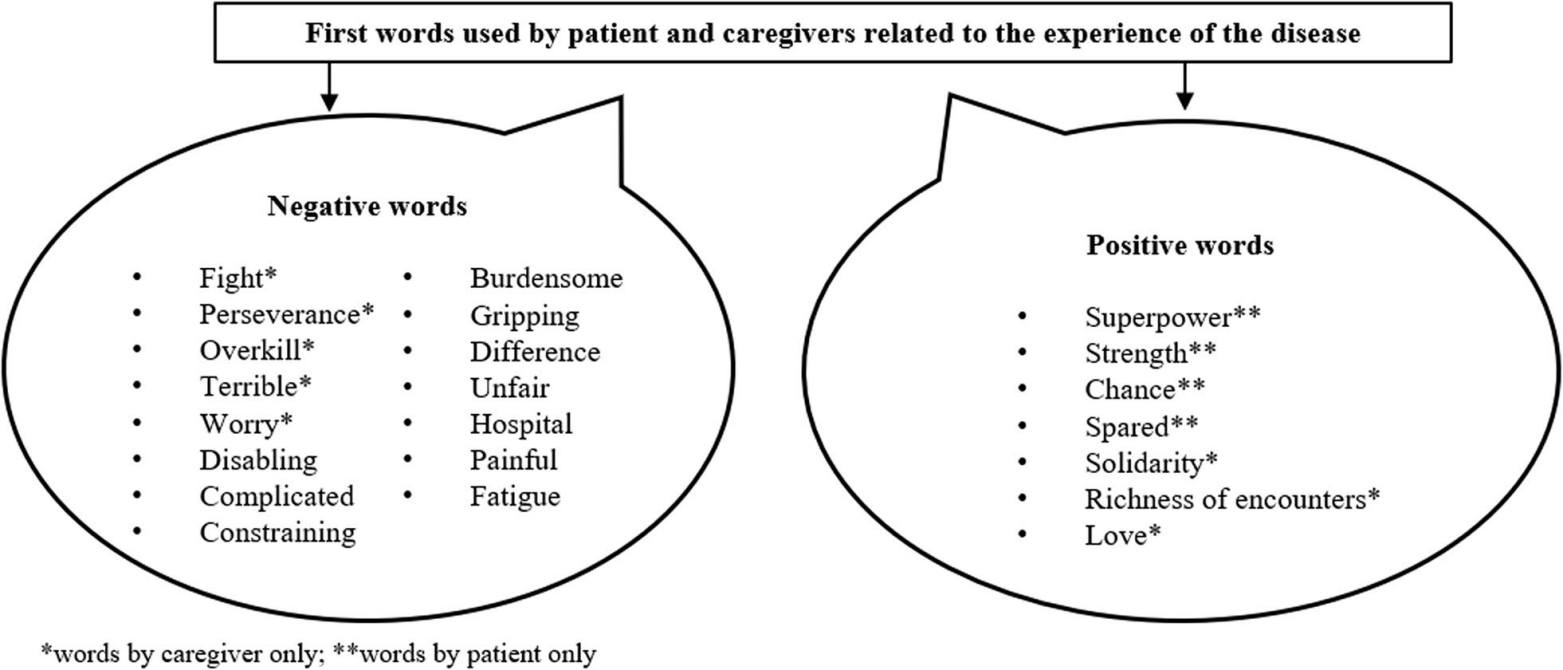
First words used by patient and caregiver related to the experience of the disease

The interviews of the responder pair (patient plus caregiver) were found to be complementary, with both caregiver and patients sharing common viewpoints about the management of the disease. The first words used to describe the experience related to the disease, either in a positive or negative tone, by patients and caregivers are presented in Fig. 1. However, patients and caregivers expressed different aspects regarding their experience with the disease. Patients were more expressive about their current medical condition, treatment benefits (if any), and impact on their daily lives, whereas caregivers were better able to comprehend the history of the patient, such as significant medical events in the patient's life, or difficulties faced during childhood.

#### Knowledge gained at the time of diagnosis

**Fig. 2:**
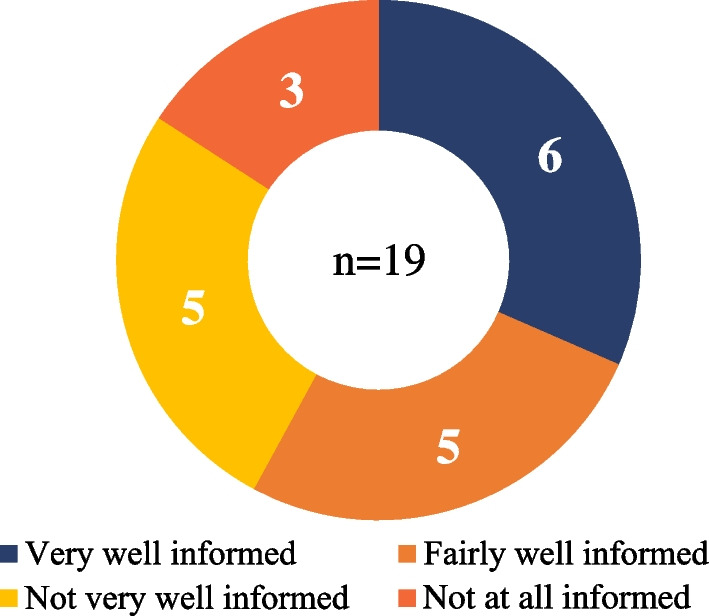
Level of information to the caregivers at the time of diagnosis

The opinions about the disease information received at the time of diagnosis were highly segmented (Fig. 2). Overall, more than 50% of the caregivers surveyed felt that they were ‘very well’ or ‘fairly well informed’. The sources of information reported by patients and caregivers were the specialists at the centre (Inherited Metabolic Reference Centre), patient association, other parents/patients, and the internet. The level of satisfaction with the information provided was based on the time taken to refer to a specialist for diagnosis, and most importantly, the quality of announcement of diagnosis. Some caregivers felt upset about the announcement about life expectancy and reported it as evasive or cold (*"Very harsh words”; “I was told that he would not live more than 5 years”)*. Moreover, the caregivers of patients with delayed diagnosis felt alone, had anxiety-provoking thoughts, lacked guidance and psychological support to prepare themselves for the reality of the disease. Currently, patients and their caregivers use words such as *“missing enzyme” “lysosome” “waste accumulation” “degenerative or progressive” “genetic” “rare”* to describe the disease, which indicates that they are now familiar with the disease.

#### Levels of disability

The degree of disability varies considerably from patient to patient and the impact of severe cognitive impairment versus non-cognitive impairment forms on the lives of patients and caregivers was incomparable. To better understand the impact on life among these two forms, patients were classified into three groups as per their level of disability: Group A, Patients not able to answer the survey question because of a severe cognitive impairment (n_p_ = 8; 3 MPS I / 5 MPS II; mean age, 23 years); Group B, Patients able to answer the survey question with low or no cognitive impairment and high motor disability (n_p_ = 10; 6 MPS I / 3 MPS II / 1 MPS VI; mean age, 33 years); and Group C, Patients able to answer the survey question with low or no cognitive impairment and low motor disability (n_p_ = 7; 2 MPS I / 1 MPS II / 4 MPS VI; mean age, 31 years). These three groups were assessed for impact of disease on their daily lives based on a scale of 0 to 10 (Table 2). It was observed that the negative impact of the disease was the highest for caregivers in Group A (Table 2). The professional activity of patients was most significantly affected in Group B versus Group C (7.8 versus 1.0) (Table 2). Moreover, the use of different orthopaedic equipment, hearing aids, corneal transplant, and medications was almost similar between Groups A and B and least in Group C (Table 3).

**Table 2:** Comparison of levels of disability, autonomy, and impact on lives between three groups

Patient characteristics	Group A	Group B		Group C	
	Caregivers (n_c_ = 8)	Caregivers (n_c_ = 9)	Patients (n_p_ = 10)	Caregivers (n_c_ = 2)	Patients* (n_p_ = 7)
*Patient's disability (0* = *no disability / 10* = *severe disability)*					
Compared to all patients suffering from the disease	6.7	5.8	6.1	4	2.8 (2)
Compared to all young people of their age	8.8	8.3	7.8	7	5.5 (5)
*Patient's autonomy (0* = *fully autonomous / 10* = *no autonomy at all)*					
Compared to all patients suffering from the disease	7.7	4.2	4.4	1	1.2 (0)
Compared to all young people of their age	8.5	7.1	6.4	3	1.4 (2)
*Impact of disease (0* = *no impact / 10* = *heavy impact)*					
Family life	7.6	6.6	6	5	0.6 (0.5)
Professional life	4.7	5.4	7.8	5	1.0 (1.5)
Social life	6.1	4.2	5.9	4	2.8 (3.5)

*n*_*c*_ Number of caregivers, *n*_*p*_ Number of patients

^*^Values in brackets represent the results from patients who were interviewed in pairs with their caregivers

Group A, Patients not able to answer the survey question because of a severe cognitive impairment (n_p_ = 8); Group B, Patients able to answer the survey question with low or no cognitive impairment and high motor disability (n_p_ = 10); and Group C, Patients able to answer the survey question with low or no cognitive impairment and low motor disability (n_p_ = 7)

**Table 3:** Difference in use of equipment between three groups

Patient characteristics	Group A (n_p_ = 8)	Group B (n_p_ = 10)	Group C (n_p_ = 7)
*Use of Orthopaedic equipment*			
Manual wheelchair	5	3	–
Electric wheelchair	2	3	–
Walker	1	3	–
Canes, crutches	1	4	–
Shell wheelchair	2	–	–
Medical bed	2	–	–
Orthopaedic insoles	1	3	3
Splint	1	3	–
Brace	2	–	–
*Hearing aids and corneal transplants*			
Hearing aids	5	4	2
Corneal transplant	2	3	1
*Previous or current treatment*			
Painkillers	8	8	3
Antiepileptics	5	1	1
Neuroleptics	3	1	–
Antidepressants	2	5	–
Anxiolytics	4	4	1
Sleeping pills	2	2	2

*n*_*p*,_ Number of patients

Group A, Patients not able to answer the survey question because of a severe cognitive impairment (n_p_ = 8); Group B, Patients able to answer the survey question with low or no cognitive impairment and high motor disability (n_p_ = 10); and Group C, Patients able to answer the survey question with low or no cognitive impairment and low motor disability (n_p_ = 7)

### Impact on lives of patients and caregivers with severe cognitive impairment (Group A)

#### Difficulties related to autonomy

In general, caregivers reported ‘loss of autonomy’ as the most tedious aspect to be managed. About three of the eight patients (< 25 years) from Group A, with a very advanced form of MPS II had degenerative changes reported as loss of mobility and verbal communication (“*He walked until the age of 13, he no longer walks, he is bedridden and I am the one who moves him in his wheelchair, he has no motor skills in his arms and legs”*). In addition, these patients had respiratory and sleep disturbances, as well as feeding difficulties, compelling the caregivers to switch to enteral nutrition. The other five patients (MPS I, n = 3; MPS II, n = 2) aged 19–30 years had reduced mobility due to stiffness and joint pain and used walking aids, displayed varied autonomy in day-to-day activities, such as external travelling for appointment visits, and reported difficulty in social interaction (*“He expresses himself, but he has learning difficulties, so he does not articulate well and does not always understand instructions”*).

#### Difficulties related to medical-educational facilities

Caregivers described schooling in a regular environment as a challenge (*“He was labelled disabled, he was marginalised”)*. Patients had to leave the mainstream school after primary level and only three of the eight patients were directed to private learning support (Table 1). Caregivers expressed their disappointment on not being referred to a suitable medico educational institutes in time that caters to the needs of polyhandicap (motor and cognitive disabilities) patients. Additionally, caregivers were also dismayed with poorly adapted learning, and lack of nursing and educational staff for MPS patients. After patients crossed the adolescence stage, caregivers had to search for adult care facilities, and only a minority (n = 2/8) of patients were able to obtain a place in nursing or special care homes.

#### Other difficulties affecting caregivers

Caregivers felt that patients have a very strong relationship of ‘dependence’ with them, especially for those taking care of the patient alone. The caregiver’s life is organised around patient care, leading to physical and psychological exhaustion (*“He needs to be taken care of all the time"; “Everything revolves around the disease”*). There was a predominant sense of insecurity and powerlessness among caregivers, owing to the painful nature of the disease (*“The most difficult thing is to see when he is in pain, and you can't help him****”***). The impact of the disease was also significant on the relationships of the caregivers; five of the eight caregivers admitted that they experienced separation from their spouse due to the disease. Notably, the impact was negative not only on relationships but also on social life and on the patient’s sibling (“*My other boy has been through these years of hospitalisation, of hassle, he took his first steps in a hospital*”).

MPS I also had an impact on the career choices of some caregivers, as they either had to stop working or switch to part-time jobs, to take care of the patients (Table 1). On the other hand, caregivers also acknowledged that professional activity was the only way to "escape", or to "breathe" their moment of respite. All caregivers in Group A felt that the administrative procedures to obtain financial support, home help, medical equipment reimbursements were burdensome, and they often had to fight with the social security centres without always succeeding.

### Impact on lives of patients and caregivers with low or no cognitive impairment and high motor disability (Group B)

#### Difficulties related to autonomy

The most challenging aspect to be managed for both patients and caregivers was the loss of mobility and chronic pain described as ‘omnipresent’ resulting in ‘restricted walking range’ (*"Beyond 150 m to 200 m, it's the wheelchair " [patient]*). However, patients exhibited variable thresholds for pain and majority of them have accepted it as a part of their life (*With our pathology, we have to live with pain constantly, daily" [patient*]). Pain strongly impacted patient’s quality of life (QoL) due to lack of suitable pain-relieving treatment, with few patients reporting that even morphine was insufficient to manage pain.

In particular, MPS I and VI patients described their bodies as ‘deformed’ and ‘robotic’ and required walking aids. Autonomy in daily activities was greatly affected by joint pain, joint stiffness, and contractures (*“It's the use of my hands that is most disabling. Using my hands is complicated, partially because I have no feeling in my fingers. Tying my shoes is torture, writing is almost impossible because it is too painful” [patient]*). Some patients were able to drive, allowing them a certain degree of independence.

Patients were equipped with hearing aids and corneal transplants to compensate for the significant impairment of hearing and corneal opacity (Table 3). Patients (n = 2) also reported the use of techniques nocturnal non-invasive ventilation (NIV) during sleep to assist breathing.

#### Difficulties related to education

‘School absenteeism’ was one of the major challenges reported by the caregivers due to patient’s multiple medical/surgery appointments. Unlike Group A (Table 1), some patients in Group B (n = 10) were able to complete their studies at university level (four-year education after high school [Bac + 4], n = 2; three-year education after high school [Bac + 3], n = 1; two-year education after high school [Bac + 2], n = 2), while others had to discontinue earlier than desired due to hearing or visual impairment. On the contrary, this was not mentioned as a challenge in Group C (n = 7), where majority of patients could complete their studies (Bac + 4, n = 3; Bac + 2, n = 2; Bac, n = 1). These patients had been referred to specialised educational programmes or vocational rehabilitation centres to pursue diploma courses. Patients were aware of their physical difference with peers and often suffered bullying.

#### Difficulties from a professional and psychological perspective

From the professional perspective, finding employment after apprenticeship and minor discrimination at workplace due to physical appearance were some of the challenges encountered by patients. Reportedly, only one of the ten patients interviewed is currently working in the mainstream environment. Except for the patients still in apprenticeships, other patients have either never worked or lost their jobs due to disability. The impact of the disease was highest on the professional life of Group B patients (Table 2).

Half of the patients (> 30 years) living independently were determined to maintain their autonomy and psychologically, they do not want to fit into the ‘disability box’. It is important to note that disease has a significant psychological impact since physical deterioration can occur suddenly. Hence, the caregivers worried about the patient’s mental health, but found psychological care ‘unsatisfactory’. Notably, the use of antidepressants is most frequent in this group (Table 3).

### Impact on lives of patients and caregivers with low or no cognitive impairment and low disability (Group C)

Currently, the majority of the patients felt that they are able to manage life, disease, and health independently. Patients were found to be ‘distanced from the disease’ and living almost a normal life; one caregiver even reported that their *“child neglects and underestimates the pathology of the disease”.* Some students (> 25 years) are living independently, employed, and even practicing a sports activity. However, few patients also reported challenges in motor abilities such as back and hip pain, joint stiffness, and fatigability.

They had minimal use of painkillers and patients in this group do not require walking assistance (Table 3). The only constrain expressed was travelling for weekly infusions especially for young patients, since all patients (7/7) in Group C versus Group A (4/8) and Group B (5/10) received ERT at day hospital (Table 4).

**Table 4:** Therapy received and location of weekly infusion of ERT

	Group A	Group B	Group C
Therapy received	(n_p_ = 8)	(n_p_ = 10)	(n_p_ = 7)
ERT	4	8	6
HSCT	2	2	1
ERT + HSCT	2	–	–
Location of therapy	(n_p_ = 6)	(n_p_ = 8)	(n_p_ = 7)
ERT received in Home Hospital Care	2	3	–
ERT received in Day Hospital	4	5	7

*n*_*p*,_ Number of patients

*ERT* Enzyme replacement therapy, *HSCT* Haematopoietic stem cell transplantation

Group A, Patients not able to answer the survey question because of a severe cognitive impairment (n_p_ = 8); Group B, Patients able to answer the survey question with low or no cognitive impairment and high motor disability (n_p_ = 10); and Group C, Patients able to answer the survey question with low or no cognitive impairment and low motor disability (n_p_ = 7)

### Perception of patients and caregivers related to medical management of MPS

#### Perception about inherited metabolic reference centre

**Fig. 3:**
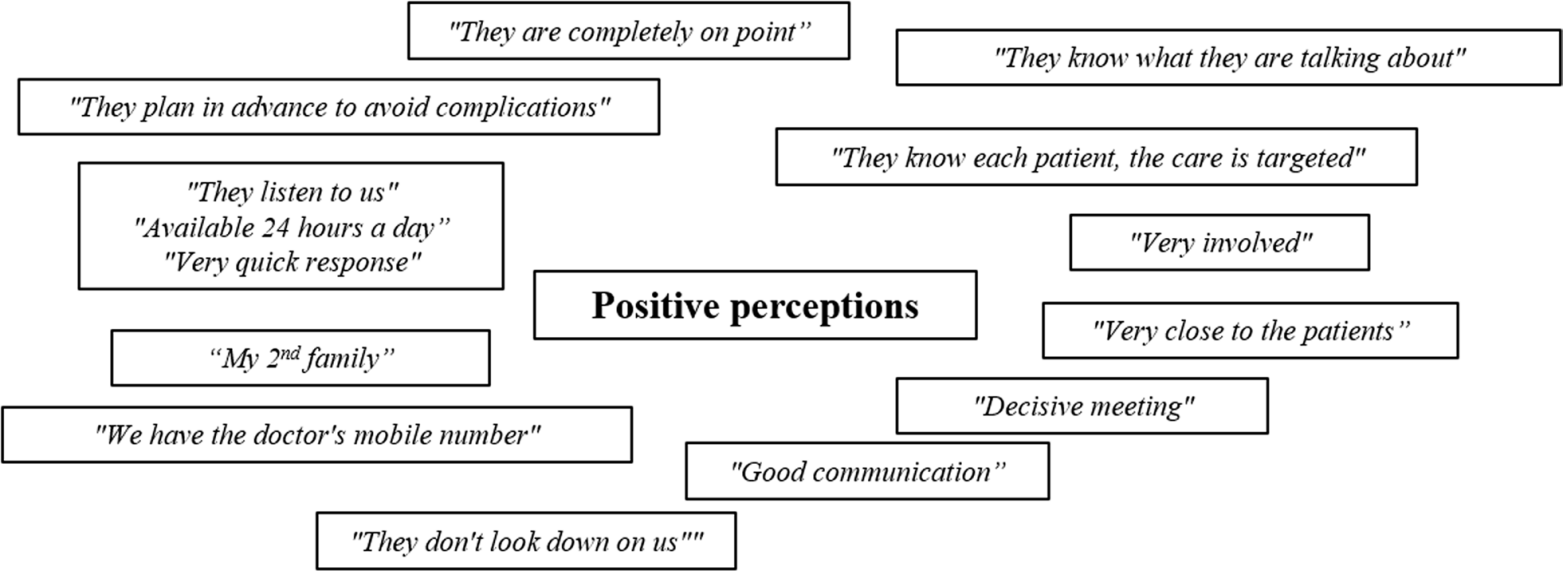
Positive perceptions of families about reference centres

The main challenge for majority of caregivers and patients is the geographical distance to the reference centre. Therefore, they only visit once or twice a year for multidisciplinary consultations (MDCs) organized by a nurse on a single day described as ‘*Marathon Day*’, which is often exhausting for the patients. Despite the distance, most caregivers preferred their reference centre for any medical procedure and had a positive opinion about it (Fig. 3).

#### Perception about surgical procedures

Surgical interventions such as HSCT, spinal decompression/lumbar spinal fusion, brain implant, cardiac surgeries, tracheotomy were anxiety-provoking and experienced by most of the patients, irrespective of disease severity. Even a minor procedure such as extraction of a wisdom tooth was described as complex *("Pulling a tooth means two days of intubated resuscitation" [caregiver]*). Despite knowing that orthopaedic procedures, particularly arthrodesis and osteotomies, immobilize patients for several months, the caregivers were in favour of these surgeries for the patient since post-surgery, patients were able to walk without a brace.

Hospitalisations and surgeries outside the referral centre were mentioned as *‘disastrous’* and *‘dramatic’*. According to patients/caregivers, doctors outside the reference centre lack knowledge regarding the disease and refuse to comply with information such as complex anaesthetic protocol provided by the reference centres. For these reasons, there were many cases of emergency repatriation of the patient to the Inherited Reference Centre.

#### Perception about the location of weekly infusions

There were mixed opinions about weekly trips for infusions; some patients reported it as ‘routine’ and a ‘relaxing moment’ (in Group A), while others found it time-consuming and are awaiting a positive response for home hospital care application. Patients acknowledged that it is imperative to follow the treatment, since it slowed down the evolution of the disease, with improvements in otolaryngology or respiratory pathologies (MPS II), joint stiffness, pain, fatigue, liver volume (reduced), and shape of the face (less coarse).

#### Perception about transitioning from paediatric to adult medicine for infusion

The majority of the patients receiving infusions were admitted to the paediatric area [(n = 8, median age: 21.5 (17–42 years)], while others were admitted to adult medicine [(n = 7, median age: 30 (21–43 years)]. Especially in Group A, both patients and their caregivers were more motivated to stay in paediatric care (*"In a children's hospital, it's more welcoming”; “I get on better with the nurses”*). Although patients were being managed in the adult medicine, they felt that transitioning from paediatrics to adult medicine was rough and abrupt (*"I had the feeling that they didn't know what to do with him, that we were a bit abandoned, we are not considered in the same way" [caregiver]; "There was no big preparation, it was more like, we're getting rid of you, good riddance!" [patient]).*

### Multidisciplinary specialities and alternative therapies

Regardless of the severity of the disease, physiotherapist (2–3 weekly sessions) was the most common professional referred for the adolescent and adult patients. Speech and language therapists, orthoptists, and psychomotor therapists were the most common speciality used by the patients during childhood.

Overall, the general practitioner had a very limited role whereas the majority of patients and caregivers required a psychologist, but they received occasional and irregular follow-ups. Alternative techniques reported to be used by patients were mainly for relaxation (sophrology, yoga) and pain (hypnosis, reflexology, magnetism, shiatsu).

### Impact of COVID-19 on MPS care

COVID-19 did not significantly impact the healthcare of patients interviewed here. Although few check-ups were delayed, no major challenges were reported by caregivers. Weekly infusions of ERT were maintained during this period and, when required, infusions were carried out at patient’s home.

### Needs and expectations expressed by all three groups of patients and caregivers

Globally, across all the three groups, the most common expectation was a more effective assessment and management of pain. The majority of psychological suffering of these patients was related to chronic pain, therefore both patients and caregivers anticipated more support from a pain relief centre or an algologist. Also, patients and caregivers wanted their rights to be recognised and looked forward to more ease in preparing administrative files for financial support from social security and health insurance. Moreover, patients transitioning from paediatric care expected proper knowledge transfer via a nurse responsible for the smooth transfer of medical information or files to adult medicine. For instance, in case of surgeries, caregivers preferred a document from reference centres for doctors (local hospitals) explaining the anaesthesia protocol. Additionally, caregivers desired easier access to specialists (odontologists, orthodontists) understanding and willing to work in this type of rare disease.

The needs and expectations of the patients and their caregivers were more related to the level of disability rather than to the type of MPS. At an early stage, both patients and caregivers expected answers for difficult questions like ‘future complications’, ‘life expectancy’, and ‘career development’. This indicated the fact that all expectations for information and support are formed at the time of diagnosis. Therefore, the best way forward is to enable patients for early referral to the most expert and knowledgeable doctors on the disease, and to local contacts (such as, patients' association).

Caregivers in Group A expect better support for suitable paediatric facilities and care teams that cater to the needs of patient’s disability. Moreover, caregivers also emphasized on strengthening and publicising the existing French platforms such as ‘Relais Handicap Rare team’ aimed at supporting the people with rare disabilities by developing the territorial network between specialized and local level. Additionally, disability clinics and nursing homes can offer caregivers specific dedicated areas for sophrology workshops, reflexology, and art therapy, so that they can relax from highly dependent relationship with patient. Similarly, caregivers in Group B expected assistance in searching specialised schools and methods of improving physical and psychological well-being for the patients. Though the patients in Group C perceived the disease as attenuated, caregivers believed that patients required ‘psychological support’ to accept the disease and not to be in a state of denial. Moreover, caregivers preferred greater flexibility in weekly infusion schedules, especially for younger patients.

## Discussion

The present survey study gathered qualitative data about the knowledge, challenges, everyday impact on their lives and overall medical management from 25 patients with MPS I, II and VI and their caregivers. For a better understanding of their needs and expectations, patients were grouped into severe and low/no cognitive impairment (high and low motor disability) groups. This survey is the first study to explore experience of French patients and caregivers across the spectrum of MPS diagnosis.

Available MPS evidence is more focussed on pathophysiological or genetic aspect of the diseases, their psychosocial facet has been not much explored [17]. Bio clinical endpoints such as urine GAG metabolites, 6-min walk test and pulmonary function tests evaluate the efficacy of various treatment modalities. However, the impact of these clinical benefits on the routine life of patients and their caregivers is not clearly understood [22]. The present survey study captures both patients and caregivers experiences from the disease diagnosis to the present, contributing to better understand their journey from childhood to adolescence and adulthood.

As today, the majority of qualitative studies conducted on MPS have included patients depending on the MPS type [15, 17, 21, 23, 24]. The present survey study included patients with MPS I, MPS II, and MPS VI since they display an overlap in many physical clinical features except intellectual development which is relatively normal in MPS VI [25, 26] This similarity in the overall clinical presentation in MPS I, II and VI may be attributed to the accumulation of common GAGs (HS and DS). Although patients with three types of MPS were included, they were further categorised based on their type of disability. To the best of our knowledge, this is the first survey study classifying patients depending on their extent of disability rather than conventional nosological classification of MPS, thereby supporting the fact that degree of locomotor disability and pain in MPS is directly proportional to the impact on life of patients and caregivers. This is line with a recent Spanish retrospective study on a cohort of adult MPS IV patients without central cognitive involvement. The burden of disease and poor health related QoL was attributed to variables such as restricted mobility, wheelchair use, pain, and limited autonomy in daily activities. These variables varied among patients since clinical presentation of MPS IV is very heterogenous [27]. Similarly, in the present survey study the major repercussions on family life and professional life of patients in Group A and B (with high motor disability) respectively, contrasts with the weaker impact observed in these areas in Group C (low motor disability) patients (Table 2).

The findings from this survey study are consistent with the previously published literature on MPS and other rare diseases with complex needs wherein mothers were reported as primary caregivers [16, 28, 29]. Similarly, 50% of caregivers were dissatisfied with the way diagnosis was disclosed. This is in line with the previous observational study conducted in France on MPS II patients [21]. Overall, chronic pain, loss of mobility, problems in daily activities, and the lack of psychological care were some of the concerns reported in all three groups, which are consistent with previous findings [30–32].

Variable degree of learning difficulties and cognitive decline is usually observed in all types of MPS. However, patients with MPS VI have no severe cognitive impairment without decline [7]. Notably, the survey study revealed that ‘school absenteeism’ due to weekly infusions or other MDCs was one of the biggest challenges for Group B and C patients.

The present survey study reported that the majority of the patients receiving weekly infusions in the hospitals and their caregivers were motivated to stay in paediatric care because of their familiarity with the paediatric setting and concern over the lack of established relationship with adult care multidisciplinary teams, especially Group A caregivers. Transitioning is a very crucial step for adolescent patients with rare diseases, to improve their QoL as adults [33]. A case series on MPS patients emphasized that patients require a standardized protocol for guiding the transition from paediatric to adult healthcare, continuity in routine evaluations throughout adulthood, and a synchronization between reference centres and local hospitals performing weekly infusions [34]. Similarly, a recent multicentre European survey study recommended that a transition coordinator should ensure a better communication between paediatric and adult healthcare systems [35]. Therefore, challenges, needs and expectations of patients and caregivers identified in this survey study can help in developing transition guidelines and specific multidisciplinary teams where paediatrician and internist can learn from each other while following up MPS patients in accordance with MPS guidelines [36].

Recently, stakeholders such as industry, regulators, and payers have become increasingly interested in obtaining insight from patient/caregiver throughout the drug development lifecycle [37]. Moreover, there is a scarcity of studies and appropriate instruments for determining the QoL in patients with MPS [38]. Apart from this, several national strategies across Europe encourage involving patients and their families in research and incorporating their voices into the policy-making for rare diseases [17]. This survey explored the challenges, unmet healthcare needs, and expectations of patients/caregivers for the management of the disease. The findings from this survey study can be used as a qualitative step to identify new clinical endpoints that can quantify the impact of a treatment modality in day-to-day life of a patient or caregiver and also as a starting point for designing specific questionnaire or patient-reported outcomes for assessment of QoL in MPS patients.

One of the major strengths of the survey study is the length, depth, and quality of these interviews. Since MPS are a group of rare metabolic disorders with only a limited number of patients reaching adulthood, only 25 patients were included in the survey study. It is noteworthy the VML patient association has a close connection with all reference centres in France. Lyon reference centre has the biggest MPS cohort and patients and their caregivers from this center were very proactive in responding to the survey. Therefore, about 23 from 25 patients included in the survey were from the same center and it can be acknowledged that this may constitute a ‘selection bias’. Also, the survey study has not explored in detail the needs and expectations of the patients in terms of treatment expectations from ERT or future treatment. Further surveys are required to assess the same among patients and caregivers. Moreover, it is also important to mention that respondents in the survey spoke about past experiences since childhood. Therefore, chances of ‘recall bias’ cannot be ignored.

## Conclusions

The survey study concluded that from the time of diagnosis to present, the most significant needs expressed by the patients and caregivers were better understanding of the disease and monitoring the complications accordingly (n = 12/17 patients and n = 16/19 caregivers), better pain management, allowing a greater number of patients to benefit from the support of a pain relief centre or an algologist (patients, n = 13/17; caregivers, n = 16/19), recognition of the rights of the patients in terms of specialized schools and ease in administrative procedures for financial assistance (patients, n = 14/17; caregivers, n = 19/19). About 21 of the total 25 patients were diagnosed before 2005, considering the development in the disease area over the last two decades, these patients would have been probably referred more quickly today. However, it is important to acknowledge that the positive perceptions reported about the reference centre suggests that the first point of contact for MPS patients in France is best in class, but still medicosocial research needs more fundings for further studies.

## Methods

### Survey study design and participants

In this survey study, information was collected from adolescent/adult patients with a confirmed diagnosis by enzyme activity testing followed by a DNA analysis of MPS I, II, and VI and their caregivers between November 2020 to April 2021 from all regions of France. Qualitative in-depth interviews lasting about 90 min were conducted by psycho-sociological moderators from an independent market research organisation specialized in healthcare field. The interview was conducted via telephone for the convenience of the patients. The survey study consisted of open and close-ended questions validated by French MPS experts and both patients and caregivers were expected to provide spontaneous responses with complete freedom of expression. The details of the questionnaire are presented in Additional file 1: Table S2. These audio interviews were recorded, followed by verbatim transcription to align it with the interviewee’s speech.

### Data processing and analysis

The responses received at the time of interview were kept confidential. The answers were analysed qualitatively [39, 40] and further aggregated with the responses of the other patients and caregivers. The results were shared with the scientific committee comprising of doctors from reference centre for hereditary metabolic diseases, along with experts in MPS and the patient association known as ‘Vaincre les Maladies Lysosomales’ (VML). Patients or their caregivers were able to access the results after the survey, by requesting the VML Association or referring physician.

### Ethical considerations

Data was collected in compliance with the regulations in force on the subject, and in particular the General Data Protection Regulation (GDPR). Only personal data necessary to serve the purpose of the study was collected. Data were protected from unauthorised access and will only be kept for a limited period of time following survey. Written informed consent for data processing was obtained from all participants before the study. Patients were given full rights to access the data, withdraw consent, express disagreement on data processing for legitimate reasons, request the rectification in case of data error, and data transfer. All responses were treated anonymously, and the consent form will be kept for evidence purposes for a maximum of 10 years after the end of the survey study.

## Supplementary Information

**Additional file 1**. Details about demographic characteristics of individual patient and questionnaire for the interview.

## Data Availability

All data generated or analysed during this study are included in this published article.

## Abbreviations

CPAP: Continuous positive airway pressure
DS: Dermatan sulfate
ERT: Enzyme replacement therapy
GAGs: Glycosaminoglycans
GDPR: General data protection regulation
HS: Heparan sulfate
HSCT: Haematopoietic stem cell transplantation
MDCs: Multidisciplinary consultations
MPS: Mucopolysaccharidoses
NIV: Non-invasive ventilation
QoL: Quality of life
US: United States
VML: Vaincre les Maladies Lysosomales
