# Supplementary material for: Understanding the challenges, unmet needs, and expectations of mucopolysaccharidoses I, II and VI patients and their caregivers in France: a survey study
Source: Orphanet J Rare Dis. 2022 Dec 23;17:448. doi: 10.1186/s13023-022-02593-2 (PMC9786416; doi:10.1186/s13023-022-02593-2)
Supplement: Supplementary file 1 — Additional file 1. Details about demographic characteristics of individual patient and questionnaire for the interview. [file 13023_2022_2593_MOESM1_ESM.docx]

**Supplementary Table S1: Demographic characteristics of all individual patients**

| **Gender** | **Age (years)** | **Current location** | **Type of MPS** | **Age at diagnosis** | **Living space** | **Specific Treatment** | **Age at initiation of 1^st^ treatment (years)** | **Paediatrics or adult medicine (location of infusions)** |  |
| --- | --- | --- | --- | --- | --- | --- | --- | --- | --- |
|  |  |  |  |  |  |  |  |  |  |
| **Group A** | | | | | | | | |  |
| M | 19 | Medical-Professional Institute/vocational rehabilitation centre | I (Severe) | 2 years | Parental home | HSCT then ERT | 2 | Paediatrics |  |
| M | 21 | Sheltered Workshop | I (Severe) | 1 year | Parental home | HSCT then ERT | 2 | Adult medicine |  |
| F | 29 | Medical foster care | I (Severe) | 8 months | Medical foster care | HSCT | 1 | - |  |
| M | 18 | Disability Clinic | II (Severe) | 3 years | Parental home | ERT | 5 | Paediatrics |  |
| M | 19 | Disability Clinic | II (Severe) | 3 years | Parental home | ERT | 6 | Home hospital care |  |
| M | 22 | Day care centre (2 days / week) | II (Attenuated) | 5 years | Parental home | ERT | 5 (clinical trial) | Paediatrics |  |
| M | 23 | 100% parental home | II (Severe) | 5 years | Parental home | ERT | 8 (clinical trial) | Home hospital care |  |
| M | 30 | Medical foster care | II (Severe) | 2 years | Medical foster care / parental home at the weekend | HSCT | 3 | - |  |
| **Group B** | | | | | | | | |  |
| F | 17 | Sixth form student, final year in general studies | I (Attenuated) | 8 years | Parental home | ERT | 8 | Paediatrics |  |
| F | 20 | 2^nd^ year of vocational certificate | I (Severe) | 18 months | Parental home | HSCT | 2 | - |  |
|  |  |  |  |  |  |  |  |  |  |
| M | 33 | Artist (Master’s in Art) | I (Severe) | 5 months | Parental home | HSCT | 1 | - |  |
| F | 40 | No profession | I (Attenuated) | 20 years | Lives alone | ERT | 22 | Paediatrics (referral centre) |  |
| F | 43 | Accounting secretary (mainstream) | I (Attenuated) | 3 years | Lives alone | ERT | 24 (clinical trial) | Adult Medicine |  |
|  |  |  |  |  |  |  |  |  |  |
| M | 50 | No profession | I (Attenuated) | 14 years | Lives alone | ERT | 33 | Paediatrics (referral centre) |  |
| M | 30 | Looking for a job | II (Attenuated) | 4 years | Shared flat with siblings | ERT | 17 | Adult medicine |  |
| M | 33 | Stay-at-home dad (married, 2 children) | II (Attenuated) | 2 years | Own home | ERT | 21 | Home hospital care |  |
| M | 41 | 100% parental home | II (Attenuated) | 6 years | Parental home | ERT | 25 | Home hospital care |  |
| F | 20 | 2^nd^ year student | VI (Severe) | 18 months | Parental home | ERT | 4.5 | Home hospital care |  |
| **Group C** | | | | | | | | |  |
| F | 36 | Volunteer in associations | I (Severe) | 14 months | Shared housing | HSCT | 1.5 | - |  |
| F | 31 | Work stoppage | I (Attenuated) | 30 years | Lives alone | ERT | 30 | Adult medicine |  |
| M | 27 | In employment (ordinary environment) | II (Attenuated) | 8 years | Parental home | ERT | 13 | Adult medicine |  |
| M | 21 | Master’s student | VI (Attenuated) | 14 years | Own home | ERT | 14 | Paediatrics |  |
| M | 24 | Engineering student | VI (Attenuated) | 10 years | Student residence | ERT | 14 | Adult medicine |  |
| M | 38 | Employed (ordinary environment) | VI (Attenuated) | 9 years | Own home | ERT | 35 | Adult medicine |  |
| H | 42 | Employed (ordinary environment) | VI (Attenuated) | 12 years | Parental home | ERT | 27 | Paediatrics |  |

ERT, enzyme replacement therapy; F, female; HSCT, haematopoietic stem cell transplantation; M, male.

Group A, Patients not able to answer the survey question because of a severe cognitive impairment (n_p_=8); Group B, Patients able to answer the survey question with low or no cognitive impairment and high motor disability (n_p_=10); and Group C, Patients able to answer the survey question with low or no cognitive impairment and low motor disability (n_p_=7).

**Supplementary Table S2: Questionnaire for patients and caregivers***

| **Patient Information** | |
| --- | --- |
| **Question 1** | Gender (Men/Women) |
| **Question 2** | Age (years) |
| **Question 3** | Current residence |
| **Question 4** | **Size of agglomeration (number of inhabitants)** |
|  | <2000 |
|  | 2000 to 50,000 |
|  | 50000 to 200,000 |
|  | >200,000 |
| **Question 5** | **Accommodation** |
|  | Parental home |
|  | Institution |
|  | Homeowner |
| **Question 6** | **Family situation**** |
|  | Living with family |
|  | Living independently |
|  | Living with a partner |
| **Question 7** | **Number and age of children (if any)** |
| **Question 8** | **Level of schooling** |
| **Question 9** | **Present or past employment** (Yes/No)** |
|  | Sheltered environment (vocational rehabilitation centre) |
|  | Ordinary environment (disabled worker status) |
| **Question 10** | Type of MPS (I/II/VI) |
| **Question 11** | Year of diagnosis |
| **Question 12** | **Department of care** |
|  | Pediatric department |
|  | Adult medicine |
| **Question 13** | Any other family members diagnosed with MPS (YES/NO) and their relationship with patient. |
| **Knowledge and information about disease** | |
| **Question 14** | **Level of information at the time of diagnosis or in early years** |
|  | Very well informed |
|  | Fairly well informed |
|  | Not very well informed |
|  | Not at all informed |
| **Question 15** | **What was the source of information at the time of diagnosis or in early years?** |
|  | General practitioner |
|  | Referring specialist practitioner |
|  | Nursing / paramedic team |
|  | Internet |
|  | Patient association |
|  | Other patients |
|  | Other, please specify |
| **Question 16** | Were any additional sources required such as Internet to know more about disease (YES/NO) |
| **Question 17** | **Current level of information** |
|  | Very well informed |
|  | Fairly well informed |
|  | Not very well informed |
|  | Not at all informed |
| **Disability linked with disease and impact on life** | |
| **Question 19** | Rate the degree of disability on a scale of 0 to 10 0 = no disability, 10 = severe disability |
| **Question 20** | Rate the level of autonomy on a scale of 0 to 10 0 = no autonomy, 10 = fully autonomous |
| **Question 21** | Rate the impact of disease on schooling/higher education/professional life on a scale of 0 to 10 0 = no impact, 10 = severe impact |
| **Question 22** | Rate the impact of disease on social life on a scale of 0 to 10 0 = no impact, 10 = severe impact |
| **Treatment of the disease** | |
| **Question 23** | Have you received a marrow/stem cell transplant? (YES/NO) If YES, specify age. |
| **Question 24** | Did you have enzyme therapy? (YES/NO) If YES, specify time duration. |
| **Question 25** | **Type of medications received** |
|  | Analgesics |
|  | Anticonvulsants |
|  | Anxiolytics |
|  | Neuroleptics |
|  | Antidepressants |
|  | Sleeping pills |
| **Question 26** | Have you received one or more surgical interventions? (YES/NO) If YES, specify time duration, type of procedure and age at the time of surgery. |
| **Question 27** | **Specify any other minor aids (YES/NO)** |
|  | A corneal transplant |
|  | Hearing aids |
| **Question 28** | **Did you receive the following care? (YES/NO)** |
|  | Physical therapy |
|  | Psychological monitoring |
|  | Speech therapy |
|  | Orthoptics |
|  | Psychomotricity |
|  | Other, please specify |
| **Question 29** | **Do you wear any of the following devices? (YES/NO)** |
|  | Orthopaedic insoles |
|  | Cane |
|  | Walker |
|  | Manual wheelchair |
|  | Electric wheelchair |
|  | Brace |
|  | Splint |
| **Question 30** | **Do you practice any of the following complementary alternative medicines?** |
|  | Acupuncture |
|  | Yoga |
|  | Relaxation |
|  | Sophrology |
|  | Hypnosis |
|  | Other, please specify |
| **Expectations raised by patients and caregivers  (Very significant/Significant/Non-significant)** | |
| Better understanding the disease and its evolution | |
| Knowing and monitoring the complications of the disease | |
| Being autonomous with the treatments | |
| Knowing how to talk about the disease | |
| Managing pain | |
| Genetics, screening, and family history | |
| Getting patient rights recognised | |
| Coffee and chat: living with a severe disease | |
| Retraining for a new occupation | |
| Other, please specify | |

*The questions were similar for both patients and caregivers. In case of patients with severe cognitive impairment with significant disability, caregivers had to enter the responses on behalf of patients and specify their relationship with the patient before filling responses in the questionnaire.

**If patient is >18 years of age.
